# Effects of Classical Breathing Exercises on Posture, Spinal and Chest Mobility among Female University Students Compared to Currently Popular Training Programs

**DOI:** 10.3390/ijerph19063728

**Published:** 2022-03-21

**Authors:** Éva Csepregi, Zsuzsanna Gyurcsik, Ilona Veres-Balajti, Attila Csaba Nagy, Zoltán Szekanecz, Sándor Szántó

**Affiliations:** 1 Department of Physiotherapy, Faculty of Public Health, University of Debrecen, 26. Kassai Str., 4028 Debrecen, Hungary; balajti.ilona@sph.unideb.hu; 2 Department of Sports Medicine, Faculty of Medicine, University of Debrecen, 12. Nagyerdei Park, 4032 Debrecen, Hungary; gyurcsik.zsuzsanna@med.unideb.hu (Z.G.); szanto.sandor@med.unideb.hu (S.S.); 3 Department of Interventional Epidemiology, Faculty of Public Health, University of Debrecen, 26. Kassai Str., 4028 Debrecen, Hungary; nagy.attila@sph.unideb.hu; 4 Department of Rheumatology, Faculty of Medicine, Institute of Medicine, University of Debrecen, 98. Nagyerdei Boulevard, 4032 Debrecen, Hungary; szekanecz.zoltan@med.unideb.hu

**Keywords:** breathing exercises, spinal and chest mobility, posture, female university students

## Abstract

Worldwide, university students’ physical health and posture are declining due to a sedentary lifestyle. The aim of our study was to evaluate the effectiveness of physiotherapeutic breathing exercises on posture and spinal mobility among healthy female university students compared to other training methods. Sixty-one female students of the University of Debrecen were assigned to breathing exercise (BE; *n* = 15), yoga (Y; *n* = 16), Pilates (P; *n* = 15) programmes and interval-training (IT; *n* = 15). Each training session lasted one hour, performed twice a week for 7 weeks. Students were assessed using standardized clinical tests. All programmes resulted in significant improvement in chest expansion. Results of Schober’s test showed substantial improvement using BE (*p* < 0.05), Y, P (*p* ≤ 0.01) programmes. Significant changes in occiput-to-wall distance (Y, P *p* ≤ 0.01) (BE *p* ≤ 0.001) were observed in three groups except the IT group. Fingertip-to-floor test (Y, P *p* < 0.05) results showed significant changes in two groups. The most outstanding effects on lateral flexion were achieved using BE (right, left *p* ≤ 0.001) programme. A comparison with results achieved using yoga and Pilates revealed that the physiotherapeutic breathing exercise programme is an equally effective method to significantly improve spinal mobility and correct postural problems in healthy young women.

## 1. Introduction

A sedentary lifestyle is causing university students’ physical health to decline worldwide [1]. The World Health Organization’s (WHO) recommendation (2020) for healthy adults between 18–64 years of age is at least 2.5–5 h of moderate-intensity aerobic type physical activity or at least 75 min to 2.5 h of vigorous-intensity aerobic type physical activity weekly to prevent the consequences of sedentary behaviour [1]. According to WHO’s definition, sedentary behaviour means any waking behaviour characterized by an energy expenditure of 1.5 METs or lower while sitting or lying (e.g., office work, driving, watching TV in leisure time, occupational or total time) and screen time means time spent watching screen-based entertainment in sitting or lying position [1].

A sedentary lifestyle is common in the general population of developed countries and even more so among university students. Based on a meta-analysis published by Castro et al. [2], university students spend 7.29 h per day sitting, based on self-reported data. The results suggest that higher levels of sedentary time are observed among university students compared to young adults in general, and this ratio has increased over the last 10-year period [2]. Similarly, Lee E. et al. [3] found that among Korean university students the mean sitting time was 7.96 h per day, and it was also shown that their stress, anxiety and depression significantly worsened if sitting hours increased [3]. According to a study by Nikitara K. et al. [4], based on Eurobarometer 2017 data, approximately one-third (36.2%) of adults under 65 were physically inactive in 28 countries in Europe. Subjects with increased sedentary behaviour had higher risks for obesity, cardiovascular disease, diabetes, cancer, hypertension, osteoporosis and osteoarthritis, compared to those who sit less [4]. Moreover, sedentary behaviour prevalence in European adolescents has not changed for 15 years. Data from the Sport and Physical Activity EU Special Eurobarometer reveal that the prevalence of a sedentary lifestyle between 2005 and 2017 remains similar (74.2 to 76.8%; *p* > 0.05). A duration of 4 h and 30 min of sitting time was determined as sedentary behaviour [5]. 

Some of the consequences of sedentary behaviour are postural disturbances [6]. Functional imbalance in muscles chains, limited chest expansion and joint range of motion can be experienced due to long-term sitting periods and a sedentary lifestyle [6,7]. The physiological curves of the spine, tilting of the pelvis and joint axes could be influenced by sedentary behaviour [8,9]. The developed muscle imbalance, called upper and lower crossed syndrome, can determine the whole posture of the body, increasing and maintaining tight and weaken muscle conditions [9,10]. The abnormal curves can inhibit stabilization of the core muscle system, significantly reducing mobility of the chest, leading to decreased deep breathing [9,10]. The muscle imbalance can be a higher risk for functional musculoskeletal complaints, pain and indirectly lower cardiorespiratory exercise tolerance among young adults [4,7].

Targeted exercises might influence these postural muscle changes [8]. According to the literature, men and women may respond to loading similarly because the cellular mechanism that regulates the physiological and biomechanical answers to exercises are the same, but there are some specific aspects in female participants, e.g., the menses cycle, which can influence their performance. According to a study by Bakar et al. [11], although untrained men and women may respond similarly to weight training, there are significant differences in baseline conditions—flexibility and strength—between genders [11,12]. 

Functional postural disturbances could be corrected by target exercises [8]. Improved flexibility can increase movement efficiency and may decrease the risk for musculoskeletal injuries [10]. Improved chest expansion by the controlled stretching and strengthening breathing exercises may play a role in preventing respiratory problems and achieving a higher level of cardiorespiratory load ability [12,13]. 

Several studies have suggested that, due to relaxation techniques, slow and controlled movements combined with deep breathing, yoga and Pilates are effective in the treatment of sedentary behaviour-induced musculoskeletal complaints, stress level and anxiety [14]. Pilates is a complex motion which contains the anatomical knowledge of “West” and the movement culture of “East”. The Pilates method, designed by Joseph H. Pilates, has 6 principles: strengthening and stabilising the centre of the human body, improving the breathing techniques, concentration, mind control, flowing the exercises into each other, and a slow and correct exercise performance [15,16]. Yoga is not solely a physical activity; it is a special philosophy of life containing traditional elements of Hinduism such as moral and ethical precepts [17]. It is a combination of physical, mental, and spiritual practices or disciplines from ancient India [18,19].

Several studies have examined the reliability and validity of the clinical tests which were used in our study, such as Schober’ test [20,21], occiput-to-wall distance (OWD) test [22], fingertip-to-floor test [23,24], spinal side bending (lateral flexion) test [25,26] and measurement of chest mobility [27]. These studies suggested that these tests are able to provide reliable information about spinal and chest mobility. The benefits of these physical tests are that all of them can be used in all circumstances, can lead to results quickly, can be carried out easily, and are simple and cheap. The studies also indicated that the results of these tests show a close correlation with results provided by medical devices, and assessments can be performed with minimal infrastructure and human resources [28,29].

Breathing exercises supervised by physiotherapists as an effective method of respiratory physiotherapy have high priority in the rehabilitation of pulmonary diseases and in the improvement of low loadability and limited mobility [30,31]. Nevertheless, there is not enough information available about the effectiveness of physiotherapeutic breathing techniques in the primary prevention of sedentary lifestyle-induced musculoskeletal and associated complaints.

The primary aim of this study was to evaluate the effectiveness of physiotherapeutic breathing exercises (BE) with regard to posture, range of motion of intervertebral joints and muscle flexibility in female university students compared to the effects of the reliable and popular yoga, Pilates and aerobic interval training as special motion programmes. Our secondary aim was to examine whether dynamic type aerobic exercises can compensate as effectively for sedentary behaviour-induced muscle imbalance and decreased flexibility as assessed by three slow motion programmes. Finally, we aimed to examine whether the ratio of sedentary behaviour in physiotherapy students is similar to relevant literature data. 

We hypothesized that BE can deliver similar improvements in terms of postural deviations and muscle flexibility as the assessed yoga, Pilates and aerobic interval training programmes. On the other hand, in our opinion, therapeutic exercises carried out in a dynamic form are not so effective in improving flexibility. Finally, we also hypothesized that the ratio of sedentary lifestyle among university students in physiotherapy is less than among university students around the world in general. 

## 2. Materials and Methods

### 2.1. Participants

In the present study, we examined full-time undergraduate female students at the Department of Physiotherapy, Faculty of Public Health, University of Debrecen (UD), who voluntarily participated after an online invitation and were randomly assigned to one of four short-term training programmes, the breathing exercise (BE) programme and three other programmes, yoga (Y), Pilates (P) and dynamic aerobic interval training (IT). 

The participating students could not have any diagnosed spinal or other musculoskeletal problems, internal organ or cardio-respiratory diseases, including asthma. These exclusion criteria were assessed based on self-reporting before the intervention. Further exclusion criteria were any symptoms that might have influenced the results of the study, such as body mass index over 29.9 kg/m^2^, abnormal fat mass around the area of thoracic and lumbar spine, pain or inflammation of joints and unstable standing capability due to pain. Participants could not participate in any of the training programmes known to and/or practised by them previously. Students with knowledge of breathing physiotherapeutic techniques could not participate in the targeted BE programme.

In order to exclude the influence of other training methods, the students were not allowed to take part in any other type of training during the assessed period. More than one absence resulted in that participant not being permitted to continue the programme. The students had to be willing, available and able to perform the assessments and tests at the specified time. Failure to participate in follow-up (participant did not complete a sufficient number of intervention training sessions or did not return for post-intervention testing) and lack of signature on informed consent resulted in exclusion from the study.

Providing elective subject credits was applied as motivation for participants in order to avoid absences, dropping out and achieve active conscientious participation of the students. In each group, a small number of participants were chosen because this is how motion programmes can be effectively executed. The chosen motion programmes required strict control by the instructors. The different techniques, positions and motions had to be corrected continuously in order to achieve the aimed development. We found that a physiotherapist and/or coach can work effectively with a maximum of 20 participants during training. All of the applied clinical tests were similar physical examination tests in our study, and according to Wiyanad A. et al. [22], the sample size calculation indicated that the study required at least 14 participants in case of OWD test for a reliability study. According to the study of Perret C. et al. [24], because the fingertip-to-floor test has excellent validity, reliability, and responsiveness, it can be used in clinical practice and therapeutic trials. The author suggested that despite the small sample size (ten patients), this simple clinical measure is very closely related to X-ray measures. A priori sample size calculation was performed based on these relevant studies [22,24].

All of the physiotherapist students of all academic years of the Department of Physiotherapy (*n* = 280) were invited, each being sent an online invitation, and they could apply for the programme voluntarily. It was examined whether the inclusion criteria were fulfilled, and there were no exclusion criteria. The previously designed number of students in each programme was 20, but it was decreased in each group according to inclusion and exclusion criteria. Those students who were not eligible could not participate in the different motion programmes. The eligible students could be randomly selected in any further motion programmes which were neither known to nor practised by them previously. The motion programs were colour coded and the students were number coded. Numbers and colours were selected by a draw. 

**Figure 1:**
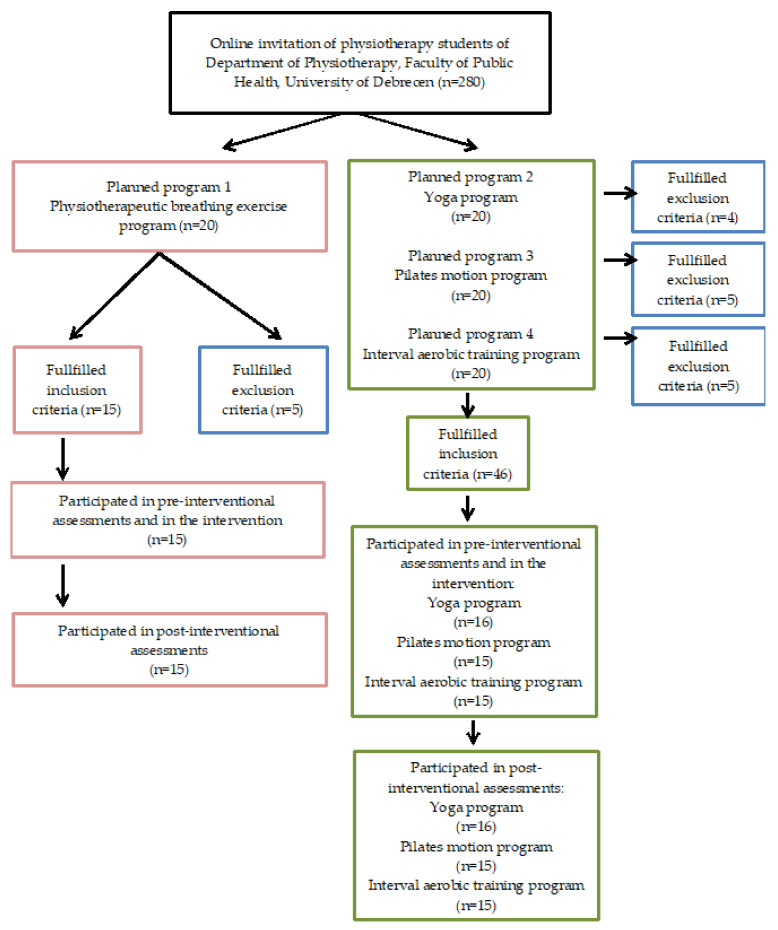
Flow diagram of subject selection process of the study.

A total of 61 females (aged 20–22 years) participated in this study (Figure 1). The BE (*n* = 15) mean age 20.1 ± 0.99, Y (*n* = 16) mean age 20.4 ± 1.40 and P (*n* = 15) mean age 20.3 ± 1.20 groups had similar anthropometric mean values, there were no significant differences between them except for the IT group (*n* = 15) mean age 21.2 ± 1.21, which differed from the other groups in body mass index and body fat rate (Table 1).

The four programmes were conducted simultaneously but on different days of the week. All assessments were carried out at the same time of day, before and after the training period. Body fat and BMI were calculated only before the intervention using the OMRON Body Fat Monitor BF306 type handle device. The individual data such as height, weight, gender and age of participant were set by the examiner in the device. The BMI and body fat were assessed in standing position keeping the device by the participant at shoulder level in front of the body during the calculation. 

The number of sitting hours and the ratio of physical activity were calculated based on self-reporting, as in the literature [2], only before the intervention. The self-reported data collection was used by oral questioning in our study. The ratio of physical inactivity was determined based on those students who had not performed any sport activity at all in their spare time in the previous one-year period.

### 2.2. Outcome Measures by Standardized Clinical Tests

The applied clinical tests aimed at providing information about posture, spinal and chest mobility before and after the training period. All tests were measured by the same experienced physiotherapist (first author), assisted by two further physiotherapists in controlling the measurements in order to avoid measurement errors. 

#### 2.2.1. Assessment of Chest Expansion

The participants were asked to be in standing position with their arms along their body. The participants being examined were asked to stand in a stable position while the circumference of their chest was measured at the level of the axilla as lower chest expansion shows a higher measurement error than the upper [27]. The tape was slowly encircled around the chest and the position was continuously under control during measuring to avoid measurement error. The participant was asked to inhale slowly through the nose and to expand the lungs as much as they could. In the second phase, they were asked to exhale the air completely through the mouth. The participant had to stay in apnoea for 2–3 s to allow for measurements by the examiner. The measurement was taken at the maximum point of exhalation and inhalation. The difference between the two rates was calculated. The assessment was repeated one more time and the better rate was used. The greater the mobility of the chest, the greater the difference. The average value of chest expansion for a healthy adult is over 3 cm [10,32]. 

#### 2.2.2. Schober’s Test

This test provides information about the range of motion of flexion of the lumbar spine. The participants were asked to stand in an upright position. After the palpation of posterior superior iliac spine on both sides, the level of spinous process of the S2 vertebra was marked and with a measuring tape a 10 cm distance upward was measured and also marked. In order to avoid measurement error, the participants were asked to bend forward slowly as far as they could with straight lower limbs without tilting their pelvis. The distance between the two marked points was measured again. We can obtain an estimate of lumbar spine flexion by calculating the difference between the rates of the two measurements. Normally the distance is 15–17 cm so lumbar flexion is 5–7 cm. If the rate is lower than normal, this can be a consequence of the decreased flexibility of the paravertebral muscles and limited range of motion of the intervertebral joints in the lumbar region [32,33].

#### 2.2.3. Occiput-to-Wall Distance Test (OWD)

This test provides information about the posture of the participants by measuring the position of the head and neck to the trunk, a simple tool for screening and monitoring to determine the presence of thoracic hyperkyphosis [22]. The results are influenced by the rate of spinal curvatures in sagittal plane; thus, OWD is also affected by the rate of thoracic kyphosis and lumbar lordosis. 

In an optimal situation the occiput touches the wall while the participants stand with their head in a neutral position. If it does not touch the wall, the distance between the wall and the occiput is measured. In order to avoid measurement error, the participants were under strict control while standing with their back against a vertical surface (wall), their heels and buttocks touching the wall in their normal standing position. The participants were standing with their head facing forward and their knees extended as much as possible. The distance between the occiput and the wall was measured by a tape. The measurement was repeated three times with a period of necessary resting. The best of the three measurements was used for the data analysis. A distance below 4–5 cm between the occiput and the wall is normal. Pre-positioned posture is defined as an OWD > 5.0 cm. If the distance is larger than normal, it can be a consequence of increased thoracic kyphosis or a head thrust forward which is due to increased lumbar lordosis. Decreased distance represents improvement of this parameter [34].

#### 2.2.4. Fingertip-to-Floor Test

This test can be used to measure flexibility of the hamstring muscle group and mobility of the thoracic and lumbar spine. Participants were asked to bend forward as far as they could with their stretched and closed knees, and to try to touch the floor slowly under strict control to avoid measurement error. Optimally the participants were able to touch the floor with their middle fingers. If they could not, the distance between the tip of the middle finger and the floor was assessed. Decreased distance means improvement of this parameter [32,33].

#### 2.2.5. Trunk Side Bending (Lateral-Flexion) Test

The rate of trunk side bending, the harmony of motion and symmetry between both sides are measured using this test. For the assessment of trunk lateral flexion, the participants were asked to stand with their backs against a vertical surface (wall), heels and buttocks touching the wall. They were asked to slide down their hands along their thighs during bending to the left and then to the right side. In order to avoid measurement error, they were under strict control during measurement when they were asked to perform large trunk lateral flexion slowly but as much as they could to the right and then to the other side without rotating their trunk forward and elevating their heels from the floor on the opposite side during trunk lateral flexion and they had to keep their shoulders against the wall. 

The distance between the tip of the middle finger and the floor was measured in lateral flexed position. There is no normal value. Decreased distance means improvement of this parameter. The test was taken on both sides. The difference between the rate of right and left side lateral flexion was also analysed. If it is larger than 2 cm, it may be due to functional deviations (e.g., quadratus lumborum muscle imbalance) or structural deformities (e.g., scoliosis) in the background [32,33].

#### 2.2.6. Heart Rate Monitoring—Pulse Control

It was necessary to carry out pulse control only during the IT fitness training in order to keep the participants’ heart rate in their own zone during the training. It was necessary to keep the pulse under control because the participants’ endurance level was also improved due to the IT programme, but the results of improvement of endurance were not presented in this study. Load intensity can be determined individually and previously using modified Karvonen formula ((220 − age) − resting pulse) × targeted intensity (between 60–80%) + resting pulse) and can be controlled during the training through pulse control. Adequate “own-zone” loading (a safe individual exercise zone) and individually planned target heart rate training zone may be achieved due to the above methods. The rate of general thresholds can be changed individually. The participants measured their own pulse. The participants had previously been taught to carry out the measurement appropriately and the process was repeated and controlled before each training to avoid measurement errors, but this process is not as reliable as assessment by pulse watch. We should know that real heart frequency is slightly greater because the measured rate can be decreased during the 15 s assessment. We conducted a test to examine the reliability of HR monitoring estimated by students. The students performed 3 sets of submaximal exercises (burpees) at approximately 60%, 70% and 80% of HR max after a short warm up. The actual HR was checked only by the investigator (first author) using a Polar H10 chest-belt. Students counted their pulse for 15 s immediately after exercising at each intensity. The investigator registered the exact HR at the beginning and at the end of the 15 s period and calculated the estimated HR by multiplying the student’s result by 4. Based on these data, intraclass correlation coefficient (ICC) was determined: the ICC(2,1) 0.8372 95%CI [0.7170–0.9082] indicated good reliability. According to this result the participants were instructed to measure their radial artery pulse for 15 s and multiply the result by 4. The participants had been informed about the subjective symptoms of overloading too, so that they could recognise in time [12].

### 2.3. Applied Training Programmes

#### 2.3.1. Breathing Exercise (BE) Programme

A breathing exercise programme is a physiotherapeutic motion therapy in which respiratory muscle training, relaxation techniques, breathing techniques, e.g., deep breathing, hand controlled abdominal/diaphragmatic breathing, aimed stretching and strengthening exercises are combined with each other [30,31]. 

The 5–10 min warm-up contained relaxation techniques, a muscle pump mechanism to improve venal and arterial circulation from distal to proximal, elongation with deep inhalation combined with relaxed exhalation and sough in order to reduce stress levels in the supine position. 

The 35–40 min training phase contained special targeted exercises for stretching and strengthening the skeletal muscles of the trunk and extremities, as well as respiratory muscles in order to improve chest mobility, muscle strength and flexibility during trunk flexion, extension, lateral flexion and rotation. Deep breathing, controlled diaphragmatic breathing, slow relaxed exhalation with pursed lips technique, direct apnoea exercises for 2 or 3 s and segmental breathing techniques with hand control on chest and/or abdomen were used. Segmental breathing technique was used for the ventilation of the basic part of lung tissue with hand control. Active cyclic breathing technique was applied as a combination of the learned techniques: all phases of respiration were guided (deep inhalation, keeping the air in for 2 or 3 s, slow exhalation with pursed lips and having a deep sough at the end). Participants inhaled through their noses and exhaled through their mouths during breathing exercises. Exercises were performed slowly in different but predominantly lying or deep crawling positions. Vertical positions such as sitting or standing were only applied in 5–10% of the exercises. Supine position was used in approximately 50% of the exercises, in which stretching exercises during trunk lateral flexion and rotation were combined with respiration. Targeted strengthening for abdominal muscles was dictated in this position in order to support exhalation and strengthen inhalation. Physiotherapeutic bridge position was dictated from supine in order to strengthen the gluteus maximus muscle and stretch hip flexors especially the iliopsoas muscle by improving hip extension. Elevation of the lower limbs to our chest and knees to forehead during trunk and hip total flexion could stretch muscles on the lumbar spine especially erector spinae and quadratus lumborum fibres. The next side lying position was applied to strengthen hip abductors as synergist muscles of trunk lateral flexors. This position was used to support chest expansion and mobility during trunk rotation and lateral flexion also by stabilised pelvis and lower limbs. Prone position was used especially to ventilate the posterior basic parts of lung tissue, to stretch pectorals and strengthen the middle and lower fibres of the trapezius muscle. We could use this position to strengthen gluteus maximus directly as the synergist muscles of trunk extensors. The next positions were all fours and deep crawling positions in which full trunk expansion could be developed while stretching pectorals and lumbar extensors and strengthening thoracic extensors in order to achieve muscle balance in the shoulder girdle. Trunk rotation could be performed by using the upper limbs against stabilised pelvis and lower limbs. Anterior plank position was applied from all fours in order to isometric strengthen the core muscles. 

Breathing exercises combined with relaxation and static stretching exercises were used during the 5–10 min of cool-down phase too in order to avoid muscle soreness and achieve calm condition in sitting position. Sitting or standing positions were used only at the end of the training for stretching during bending to toes with straight knees and improvement chest mobility during guided respiration combined with trunk lateral flexion and rotation. 

The different positions could support ventilation of different parts of lung tissue and the effect of gravity on skeletal muscles was changed in various positions, which can influence the effects of the exercises. Quiet relaxing music was played during the exercises. The aims of the breathing exercises were to ventilate all parts of the lung tissue and consequently improve gas exchange by influencing thoracic-abdominal pressure due to abdominal breathing techniques and to stretch and strengthen the respiratory muscles [35,36].

Stretching exercises were used to teach participants to identify the feelings associated with tensed muscle and fully relaxed muscle and total resting condition. The physiotherapist focused on improving all types of trunk motions while the pelvis was stabilized in order to have clearly separated movements and more efficient activation of the targeted muscles. The exercises were not demonstrated but all motions were dictated in detail by the physiotherapist and the incorrectly performed movements were corrected throughout the programme. The exercises were under strict continuous control by the physiotherapist by dictation and hand control if necessary, during the whole session. The programme was aimed at achieving a more effective breathing technique and improving spinal and chest expansion using the above special breathing techniques and dictation of all phases of inhalation and exhalation too. Easier exercises were dictated 3 or 4 times at the beginning. More difficult exercises were provided, and the repetition number was increased when the participants were stronger in order to achieve progressive improvement. The breathing exercise programme was supervised by an experienced physiotherapist.

#### 2.3.2. Yoga (Y) Programme

Yoga involves special positions (poses) to strengthen the muscles and to maintain well-being; it is also a spiritual philosophy, with the guidance of the master, repetitions of mantras, the regulation of respiration, and meditation by relaxation making it a self-analysing way of life focused on perfecting one’s self physically, mentally and spiritually [37]. The 5–10 min long warm-up was started in every session with relaxation techniques combined with elongation and deep respiration. This phase plays an important role in reducing muscle tone as well as achieving total physical and mental relaxation. The instructor placed greatest emphasis on strengthening and stretching the trunk muscles in order to improve the mobility of the spine in the 40–50 min of the training part. The exercises followed each other in accordance with the principle of gradation of difficulty and concentration. The exercises were demonstrated and corrected by the instructor during the exercises. The participants had to perform the movements in conjunction with breathing. The instructor introduced the students to different yoga positions in each session, thus making the lessons more difficult. The 5–10 minutes’ cool-down phase contained relaxation in order to achieve calm condition in supine or sitting positions. 

Yoga poses were performed slowly in 50% of cases in supine, prone and all fours and approximately in 50% in vertical positions during training. The achievement of the poses was guided step by step by the instructor and easier and more difficult alternatives were provided for participants. All phases of respiration were not dictated, in contrast to the BE programme. The participants were instructed to concentrate on their respiration in their own rhythm while keeping the achieved poses. The yoga training contained predominantly exercises for the mobilisation of the trunk and pelvis muscles in order to improve chest mobility, muscle strength and flexibility. Exercises to improve balance skill were also used by decreased supporting surface during the Y programme approximately in 5–10% in contrast with the BE programme. New and more difficult poses were dictated if the participants were stronger in order to achieve progressive improvement. The yoga programme was supervised by an experienced yoga instructor. 

#### 2.3.3. Pilates (P) Programme

Stretching and strengthening the trunk and pelvis muscles combined with respiration were in the focus during Pilates exercises. The 10–15 min warm-up contained relaxation techniques combined with stretching and deep inhalation on each session. This phase played an important role in reducing muscle tone and achieving a calm condition of participants. 

The 30–40 min of the training part focused on aimed strengthening and stretching of trunk and hip muscles due to predominantly trunk flexion and extension and rotation in order to improve the mobility of the spine. The special Pilates exercises followed each other in accordance with the principle of gradation of difficulty and concentration [38]. The exercises were demonstrated and the incorrectly performed movements were corrected by the instructor during the exercises of the Pilates programme. The participants learned to perform the demonstrated movements in conjunction with breathing. The 10–15 min cool-down phase contained relaxation combined with static stretching and a deep breathing technique in order to avoid muscle soreness and achieve a calm condition.

During the performance of the exercises the basic principles of the Pilates method were in the focus. Concentrating on inhalation and exhalation was combined with relaxation and targeted strengthening or stretching exercises but special breathing techniques such as active cyclic breathing technique or sniff breathing and pursed lips exhalation techniques, etc. were not applied. Overall, 60% of the exercises were performed slowly in supine, prone and all fours and approximately 40% in sitting or standing positions during training. To achieve progressive improvement, new and more difficult exercises were dictated when the participants were stronger. The Pilates programme was supervised by an experienced Pilates instructor.

#### 2.3.4. Interval Training (IT) Program—Dynamic Aerobic Endurance Training

Interval training is dynamic special aerobic endurance training in which low- and high-intensity periods vary in a previously determined ratio [12]. In order to improve muscle oxidative and endurance capacity interval training (IT) is an established exercise method used in the rehabilitation of patients with chronic problems, in the primary prevention of a healthy population and also in the preparation of athletes [39]. 

The 10 min warm up contained dynamic stretching and aerobic type exercises in standing position in order to achieve required heart and respiratory frequency as preparation for the intensity of the work-out phase. The 35–40 min of the work-out phase was structured by low- and high-intensity periods varying in the ratio of 3:1 (6:2 min). The intensity was 65–70% of heart rate (HR) max during the active resting period in standing position and 75–80% of HR max during the high-intensity period, which was measured by the technique of pulse control. The rhythm of the music was between 133 and 136 bpm during this fitness training programme in order to keep intensity under control. In the active resting period, low-impact, low-intensity aerobic exercises were performed to improve the cardiovascular system using easy choreography in standing position. Low-impact but high-intensity exercises were carried out to improve strength—endurance in the high-intensity period. The resting period was performed together in one group while the high-intensity exercises were performed in small groups on stages. The exercises were demonstrated and the incorrectly performed movements were corrected orally by the instructor during the exercises. Similar kinds of aimed strengthening exercises were performed for the muscles of the anterior, posterior, lateral and spiral muscle chains of the shoulder girdle, trunk and pelvis girdle in the same lying and crawling positions as the ones used during the BE programme, but especially dynamically in the high-intensity periods. The 10–15 min cool-down phase contained relaxation techniques combined with static stretching and deep inhalation on every occasion in order to reduce muscle tone, avoid muscle soreness and achieve a calm condition of the participants in standing position. Approximately 30% of the exercises were performed dynamically in supine, prone and crawling positions and approximately 70% in standing position without guided respiration and dictated breathing techniques during training. More difficult exercises were dictated and the ratio between the low- and high-intensity periods was changed (2:1; 1:1) if the participants were stronger, in order to achieve progressive improvement. The IT programme was supervised by an experienced fitness instructor. 

The four training programmes were performed twice a week, one hour per occasion, for 7 weeks. No other programme was provided for them that would use breath control, in order to exclude its influence.

### 2.4. Statistical Analysis

The baseline measurements’ data are presented as mean ± SD. The standardized clinical tests’ data are presented as medians and interquartile ranges (IQR). The Shapiro–Wilk test was used to check the normality of the continuous variables. Since most of the data did not follow normal distributions, non-parametric Wilcoxon signed-rank test was used to compare medians. The Spearman’s correlation analysis was used in order to investigate the correlation between the variables related to the physical examination. The degree of difference between the four groups’ baseline condition was determined by Kruskal–Wallis ANOVA. Dunn’s post hoc test was carried out for pairwise comparison of baseline and final pairwise differences. The results were considered as significant if the *p*-value was below 0.05. The data were processed using Microsoft Excel and the statistical analysis of the data and calculation were made using the Intercooled Stata v13 programme [40].

### 2.5. Sample Size Calculation

A priori sample size calculation was performed with a power level of 80% and an α level of 0.05 based on relevant studies. [22,24] An online invitation was launched among all physiotherapist students in order to recruit the appropriate number of participants. Refusals, exclusions and group size (to ensure proper attention) were also taken into consideration. 

### 2.6. Ethics

Informed consent was obtained from all volunteering participants according to the Declaration of Helsinki [41]. This study was approved by the Institutional and Regional Ethics Committee of University of Debrecen (registration number: 4598-2016).

## 3. Results

### 3.1. Description of the Participants

The previously designed number of students in each programme was 20, but it was decreased in each group according to inclusion and exclusion criteria. More than accepted numbers of absences and dropouts were not observed during the motion programmes.

The BE, Y, and P groups had similar anthropometric mean values, and there were no significant differences between them. The IT group differed from the other groups in body mass index (BMI) (ANOVA test (*p* = 0.001), and body fat rate (ANOVA test (*p* = 0.044), but all fell into the “Normal” category (18.5–24.9 kg/m^2^) based on the mean value of BMI, and three groups were categorized into the “Healthy” (21–33%) but the IT group was categorized into the “Overfat” category (34–39%) based on the mean body fat content (Table 1). The students were from all academic years.

The number of sitting hours can be presented based on self-reporting (*n* = 61). According to the answers, the total of sedentary hours was 2 or 3 h per day in the case of 14.7% of students, 4–6 h per day in the case of 36.1% of students, and 7 h or more in the case of 49.2%. 

The physically inactive students accounted for 48.9% of the assessed 61 students. The other 51.1% had different regularity of physical activity: 36.2% of them reported regular weekly sport activity and 14.9% reported only casual physical activity. 

### 3.2. Chest Expansion Results

With respect to chest expansion, statistically significant improvements were observed in all training programmes (BE (*p* ≤ 0.001), Y (*p* = 0.003), P (*p* = 0.002), IT (*p* = 0.021) (Table 2). Chest expansion improved in 14 participants (93%) in the BE group, in 14 participants (88%) in the Y group, in 12 participants (80%) in the P group and in 9 participants (60%) in the IT group. Stagnation could be observed only in 8 participants (BE: 1 participant; Y: 2 participants; P: 3 participants; IT: 2 participants). Relapse was observed in 6 persons (Y: 1 participant; P: 1 participant; IT: 4 participants). There was no relapse in the BE group.

### 3.3. Schober’s Test Results

Statistically significant improvements were achieved in three groups, but no significant development was seen in the IT group (BE (*p* ≤ 0.05), Y (*p* = 0.002), P (*p* = 0.003), IT (*p* = 0.271) (Table 3). 

### 3.4. Occiput-to-Wall Distance Test Results

Decreased rate represents an improvement in OWD. Statistically significant improvements were observed in three groups, but no significant development was seen in the IT group: BE (*p* ≤ 0.001), Y (*p* = 0.003), P (*p* = 0.003), IT (*p* = 0.917) (Table 4).

### 3.5. Fingertip-to-Floor Test Results

Statistically significant improvements were observed in two groups (Y (*p* ≤ 0.05), P (*p* < 0.01). The rates were similar in BE group (*p* = 0.056) but did not change significantly. Relapse was observed only in the IT group (Table 5).

### 3.6. Trunk Side Bending (Lateral Flexion) Test Results

Decreased rate represents an improvement in this parameter. The difference was not greater than 2 cm between the mean rates on both sides in all groups during the assessment of side asymmetry. 

When lateral flexion towards the right side was assessed, statistically significant changes were found in two groups (BE (*p* = 0.001), IT (*p* = 0.014). The rate of improvement was not significant in the Y and P groups (Table 6).

With respect to lateral flexion to the left side, statistically significant improvements were achieved in three groups (BE (*p* = 0.001), IT (*p* = 0.019), P (*p* = 0.031). The rate of improvement was not significant in the Y group (Table 7).

### 3.7. Results of Kruskal–Wallis ANOVA and Dunn’s Post Hoc Tests

The degree of difference between the four groups’ baseline condition was determined by Kruskal–Wallis ANOVA test and Dunn’s post hoc test was carried out for pairwise comparisons (Table 8).

There was no significant difference between the four groups in the baseline condition of chest expansion before the training programmes (*p* = 0.137) and it remained unchanged as a result of programmes (*p* = 0.805) after the training period.

There was no significant difference between the four groups in the baseline condition of Schober’s test before the training programmes (*p* = 0.358) and the difference was borderline significant between the four groups (*p* = 0.050) after the training period.

There was a significant difference between the four groups in the baseline condition of OWD test before training program (*p* = 0.009), but this was the result of the significant difference between BE and IT programmes (*p* = 0.004). The difference was significant too between the four groups (*p* = 0.001) after the training period.

There was no significant difference between the four groups in the baseline condition of fingertip-to-floor test before the training programme (*p* = 0.079) and the difference was not significant between the four groups (*p* = 0.947) after the training period.

There was no significant difference between the four groups in the baseline condition of trunk side bending (lateral flexion) to the right test before the training programme (*p* = 0.105) but it was changed due to the program because a significant difference was calculated (*p* = 0.019), after the training period.

There was no significant difference between the four groups in the baseline condition of trunk side bending (lateral flexion) to the left test before the training programme (*p* = 0.343) and the difference was not significant between the four groups (*p* = 0.091) after the training period on the left side. 

### 3.8. Results of Differences between the before and after Values Related to the Four Motion Programs

Significant differences were observed between the before and after values related to the motion programs according to calculation by Wilcoxon signed-rank test (Table 9).

### 3.9. The Spearman’s Correlation Analysis Results

The Spearman’s correlation analysis was used in order to investigate the relation between the variables related to the physical examination. Three significant relations were found:

The results of Spearman’s rank-order correlation showed a small, negative but significant relation between the results of Schober’s test and the occiput-to-wall distance test (rho = −0.2716; *p* = 0.002). 

The results of Spearman’s rank-order correlation showed a small, negative but significant relation between the results of fingertip-to-floor test and the chest expansion (rho = −0.1915; *p* = 0.035).

The results of Spearman’s rank-order correlation showed a small, positive but significant relation between the results of fingertip-to-floor test and the occiput-to-wall distance test (rho = 0.3696; *p* < 0.001).

## 4. Discussion

Results suggest that BE may be an effective alternative therapy to improve posture, flexibility, strength and consequently balance of the skeletal muscles and range of motion of the intervertebral joints in healthy young adults because significant changes were achieved by BE in most assessed parameters.

Results measured before and after the interventions by clinical tests can be presented only based on assessment of the women in our study. In order to avoid influencing the outcomes and have reliable results homogenous female groups were assessed and compared to each other in our study because of the difference between genders in strength and flexibility [11,12].

It is known that a sedentary lifestyle and its negative physiological effects are civilizational hazards in developed countries, and it has been recently revealed that this physical inactivity results in shoulder, middle and lower back pain and depression, especially among women with an average sitting time longer than 4 h per day [3,4]. Sedentary behaviour is common in university students, which may be responsible for musculoskeletal complaints [1,2]. According to our results, the physically inactive students accounted for 48.9% of the 61 assessed students. Total sedentary hours were reported as 4–6 h per day in case of 36.1% of students and as 7 h or more in the case of 49.2% in our study. According to these results our third hypothesis was not confirmed because the ratio of a sedentary lifestyle was similar in the assessed students of physiotherapy to the university students around the world in general.

Czakwari et al. assessed [6] spinal abnormalities among students of the Medical University of Silesia (54 female, aged 20–28, and 46 male, aged 20–29) using modified Klapp protocol. Postural faults were widespread in the assessed group. The most common of these deformities were lumbar hypolordosis (71.0–48.1% female and 97.8% male) and thoracic hyperkyphosis (58.0–53.7% female and 63.0% male), and the prevalence of less frequent scoliosis was higher than 50% (50% female and 58.7% male). Physical activity in the assessed group was high (71–76% female and 62.5% male). The level of activity in men was significantly higher than in women (*p* < 0.05). The authors concluded that there was no correlation between levels of physical activity and postural faults according to their results. These findings partially agree with our results as well. Our findings showed that physical inactivity was widespread in our assessed group too. The ratio of physically active students in our assessed group was lower (51.1%) and if regularity is calculated, this number is even lower because 36.2% reported regular weekly sport activity.

We would like to draw attention to the fact that it is necessary to increase the number of those students who have regular physical activity, and the level of regular physical activity level should be improved by different, compulsory registered training programmes providing subject credits to university students in order to achieve and/or maintain better musculoskeletal condition among them. Providing elective subject credits was used to motivate participants in order to avoid absences and dropout and to achieve the active conscientious participation of students. In our opinion, this motivation factor played an important role, in that more than accepted numbers of absences and dropout were not observed during the motion programmes.

Our present study also proved that the flexibility of the examined female university students measured by the special tests is low. The results of the Schober’s and finger-tip-to-floor tests suggested that approximately 30% of students had limited range of flexion of the lumbar spine, and more than half of them had complex postural abnormalities reflected by an occiput-to-wall distance larger than 4 cm. Besides the postural and flexibility problems of the spine in the sagittal plain, the difference between the rate of right and left side lateral flexion in 23% of participants was larger than 2 cm, due to functional deviations or structural deformities in frontal plain. These results are in harmony with the ratio of sedentary hours and physical activity among assessed physiotherapist students. The larger ratio of muscular postural problems and limited joint mobility is a consequence of the larger ratio of sedentary hours and the physical inactivity.

The correlation analysis shows the relation between the tests’ results and can confirm the effectiveness of a provided motion program. The analysis was provided based on the studies of Viitanen et al. [42] and Heuft et al. [34]. According to our results of Schober’s test increase, those of the OWD test decreased (which means that both results show improvement). This analysis can show that if we improve the Schober’s test result, we can achieve an improvement in the rate of the occiput-to-wall distance test. If we improve the mobility of the lumbar spine, we can achieve a more optimal rate of cervical lordosis and a more physiological posture, according to the OWD test. The positive significant relation between the results of fingertip-to-floor test and the occiput-to-wall distance test are in harmony with the previous correlation too, because the decreased rates reflect improvement in both tests and the fingertip-to-floor test is carried out with trunk flexion too similarly to Schober’s test. The small, negative significant relation between the results of fingertip-to-floor test and the chest expansion can be logical too because if the results of fingertip-to-floor test decrease, those of the chest expansion increase (which means that both results show improving). The elongation of thoracic spine during touch the floor with fingertips can be the explanation. The results can be more reliable based on the harmony relation between the applied tests. Our results suggest that the used tests can confirm each other during assessment of posture and spinal mobility [34,42].

Our results suggest that specific targeted exercises are necessary to compensate for the effects of a sedentary lifestyle. Regular but not well-structured training programmes are not sufficient for correction, so we supposed that the quality of the training plays as important a role as quantity in the correction and/or maintenance of mobility and muscle balance.

The BE had surprisingly good results. The most significant improvement was achieved in chest expansion and trunk lateral flexion, while the Y programme did not result in a significant improvement in the side bending (lateral flexion) test. A significant improvement was achieved as a result of BE in Schober’s and OWD test, in contrast with the IT training, while the fingertip-to-floor test showed relapse in the IT group. These results may be explained by the structure of the training and the effects of direct and indirect effect-mechanisms of the special breathing techniques. BE were performed in different positions, in order to improve trunk and chest mobility. We dictated a number of aimed stretching and strengthening exercises for intercostal muscles, segmental breathing at the basis of the lung tissue by trunk lateral flexion and rotation on all fours and deep crawling positions. The trunk flexion was the least used motion type during BE training. This factor must account for the results of the Schober’s and finger-tip-to floor tests.

Stretching exercises for the diaphragm muscle were dictated in those kinds of positions when exhalation is performed by cut-off or help of gravity force, as in all fours, deep crawling or bridge positions. Inhalation can be dictated in all fours position and exhalation in deep crawling position if the aim is the facilitation of exhalation and stretching of the diaphragm muscle. We can use these positions the other way round too, and inhalation is dictated in deep crawling and exhalation on all fours if strengthening of the diaphragm is our aim. The all fours position is the easiest for the diaphragm muscle during respiration. The deep crawling positions are so effective for posture correction because they may positively affect the strength and flexibility of the respiratory and paravertebral muscles and consequently the spinal curves and shoulder girdle. Static isometric exercise was dictated in anterior plank position to activate the serratus muscle and deep core muscles in order to improve their stabiliser functions around the scapulo-thoracic functional attachment and spine.

The exercises of BE were chosen based on the sedentary behaviour-induced postural disturbances [6]. The changed vectors of the muscles can cause limited capability to keep optimal spinal curves against external axial compression forces [10]. The line of gravity is shifted anteriorly because of the forward tilted position of the pelvis and induces larger spinal curves due to a sedentary lifestyle [9]. Flexion is induced in the hip, extension in the knee and plantar flexion in the ankle joint occur [10]. Increased tone, limited function and weakness develop mainly in the quadratus lumborum muscle, in the paravertebral muscle fibres as in the erector spinae muscle on the lumbar spine, in the tight hamstring and iliopsoas muscle fibres [9,10]. The stabiliser cross synergist relation in sagittal plane is weakened between the lower fibres of the rectus abdominis muscle and the gluteus maximus because of their weakness and elongated condition [9]. Forward headed position occurs because of the elevated tone, especially of the upper trapezius, sternocleidomastoid, scalenes, levator scapulae and suboccipital muscles, and also pectorals, against the elongated and weak middle and lower trapezius fibres and paravertebral muscles on the thoracic spine [9,10]. Posterior tilted pelvis position can also develop, in which case the sagittal spinal curves are decreased and a more rigid structure develops [10]. Overloading occurs on tonic type muscles but decreased cross-sectional size (atrophy) can be experienced on phasic type muscles [9]. The abnormal curves cause an inhibited stabilizer function of the transversal abdominis muscle and deep spinal segmental stabilizers as of the multifidus and rotatores muscles [10]. Larger shear forces can be performed especially in the cervicothoracic and thoracolumbar regions, contributing to disc degeneration process [9,10]. The correction of muscle imbalance is in the focus during posture correction. Muscle imbalance can be influenced effectively by activating the spiral and lateral muscle chains due to applied trunk rotation and lateral flexion based on our results too. The thoracic spinal curve and chest expansion can be treated positively by activating of the thoracic extensors. Activation of the longus colli muscle is a key to the achievement of optimal cervical spinal curve. Balance around the lumbo-pelvic-hip complex can be supported positively by activating the sagittal cross synergism between the trunk flexors and hip extensors [9,10].

According to our results the positive effects of Pilates [15,16] and yoga [17,18] programmes were confirmed in accordance with the literature. Significant changes were observed in all clinical tests due to these programmes except for the lateral flexion. Exercises for improving trunk flexion, extension and rotation were more dominant during the Y and P programmes, but the scale of lateral flexion grew especially during the BE and IT programme. Physiotherapeutic breathing techniques and all phases of respiration were guided only during the BE programme.

Our results seem to confirm our secondary hypothesis. The IT programme is an effective and reliable training method primarily to improve endurance level because of various intensity dynamic exercises [12,39]. The IT training method was used because we wanted to find out whether these dynamic type trainings can compensate as effectively for sedentary behaviour-induced postural deviations as slow, controlled motion programmes. Stretching and relaxation techniques played an important role in all programmes, but in the IT programme there were fewer exercises of this type. Dynamic stretching was used during warm-up and static type during cool-down phase only. The varied results of the IT group were probably due to aimed strengthening exercises performed dynamically. According to our results the flexibility and range of motion of the joints can be improved more effectively by slow and controlled exercises than by dynamic exercises. The ANOVA test showed a significant difference between the baseline conditions in the OWD test because of the IT group, in which the median and IQR were much better than in the other groups before the programme but did not change significantly due to the exercises. The baseline condition was more homogenous in the finger-tip-to-floor test in the IT group than in the other groups, but a relapse of results was observed as a result of the programme. The IT group differed from the other groups in body mass index (BMI) and body fat rate according to the ANOVA test, which could also affect the results. The outcomes may be influenced by the previous differences between the baseline conditions, but our results suggest that exercises to improve postural problems, and correct muscle imbalance should be performed slowly under strict control by an instructor. Effective postural control and improvement in muscle flexibility cannot be achieved by exercises performed dynamically.

Other postural training interventions have also been assessed. Celenay et al. [43] examined the effects of different methods on spinal posture and mobility such as electrical stimulation, exercises, biofeedback posture trainer and postural education. They found that the exercises were more effective in terms of improving thoracic and lumbar curves, and mobility among university students. Other methods had limited effects, electrical stimulation decreased thoracic curve, while sitting posture was improved by a biofeedback posture trainer. These results suggest that exercises provided by real activation of participants’ own muscle power are more effective in improving mobility than device-assisted motions.

Our BE therapy, supervised by a physiotherapist consisted of special, slow stretching exercises to improve the flexibility of skeletal, especially respiratory, muscles and chest mobility and involved targeted strengthening combined with voluntary apnoea exercises, deep, segmental and controlled abdominal (diaphragmatic) breathing techniques with hand control and relaxation, through which directly and indirectly functional postural deformities may be corrected more effectively along with the correction of muscular imbalance. The results of this study, similarly to some previous studies, suggest that special structured, short-time training programmes, including targeted exercises, may be effective in improving chest expansion, flexibility of muscles and ROM of joint, because even with a relatively small amount of time, we can do a great deal to prevent our health by using appropriate techniques [35,36].

While different fitness training programmes, Pilates and yoga are essentially preventive movement programmes that assume physiological body structure; however, physiotherapeutic breathing techniques do not assume this (musculoskeletal system, cardiovascular and respiratory system) [30,31].

Our results confirmed our primary hypothesis. Based on our results, the breathing exercise programme, which is a targeted physiotherapist-specific motion therapy and can be used both in the secondary and tertiary prevention of patients, can be used in the primary prevention of sedentary behaviour-induced postural problems and decreased muscle flexibility too to prevent further musculoskeletal complaints and consequences in healthy young adults such as university students. According to the literature, targeted breathing exercises can play an important role in the prevention and rehabilitation of cardiorespiratory diseases especially pulmonary consequences but the targeted breathing exercise programme can not only support the recovery of patients but can also provide further development for healthy adults [44,45].

We recommend the incorporation into our daily routine of 3–5 easily performed exercises such as elongation combined with breathing exercises with trunk flexion, extension, lateral flexion and rotation in standing position during the day, and/or performance of 10–15 min stretching exercises combined with breathing techniques for the improvement of spinal flexibility and chest mobility on non-weight bearing positions at the beginning and/or at the end of the day.

In our opinion, it is also advisable to combine postural exercises with endurance training weekly in order to achieve the most effective prevention of sedentary behaviour-induced consequences [46].

## 5. Conclusions

The results show that a targeted and specially structured breathing exercise programme supervised by a physiotherapist, with slow and controlled movements, is an effective motion programme to improve posture, muscle balance, spinal and chest mobility, due to direct and indirect effect-mechanisms in healthy young female adults, in comparison with yoga and Pilates motion programmes.

Our study suggests that a breathing exercise programme can be safely used as an adjunct exercise programme not only for patients, as it may have primary preventive positive effects on the posture, flexibility and strength of healthy adults too. Physiotherapeutic breathing techniques combined with targeted stretching and strengthening exercises can be used by everyone, not only in rehabilitation but also in primary prevention as part of cross-training.

## 6. Limitations

The limitations of this study were the small number of assessed participants, limited sufficient time for the motion programs and physical examinations. The participants were randomly assigned to the motion programs, but the process of participant allocation was complex. A further limitation of this study was the lack of possibility to provide measurements by valid medical devices as, e.g., spirometer and radiography or spinal mouse device. Further randomized controlled trials are recommended, to gain a deeper understanding of the effects of such an intervention.

## Figures and Tables

**Table 1:** Anthropometric data of university students who participated in four different training programmes (mean ± SD).

Groups	Breathing Exercises (BE)	Yoga (Y)	Pilates (P)	Interval Training (IT)
Age (year)	20.1 ± 0.99	20.4 ± 1.40	20.3 ± 1.20	21.2± 1.21
Number of participants (female:male)	15:0	16:0	15:0	15:0
Body Mass Index (kg/m^2^)	20.97 ± 2.51	21.35 ± 3.42	20.89 ± 2.29	24.49 ± 2.64
Body fat (%)	30.15 ± 5.05	29.66 ± 7.50	28.29 ± 5.93	34.31 ± 4.82

**Table 2:** Results of the chest expansion (cm) of the four groups (target BE programme and three other programmes: yoga, Pilates and dynamic interval training) before and after the training period. The students participated voluntarily in and were randomly assigned to training programmes. The duration of each training session was one hour, twice a week for 7 weeks.

Chest Expansion—Axillar Level (cm)							
	before			after			
	Q1	Median	Q3	Q1	Median	Q3	*p*-Value
Breathing exercises (BE) (*n* = 15)	3.5	4.5 (min 3.0; max 7.0)	5.0	6.0	7.0 (min 6.0; max 8.0)	8.0	≤0.001
Yoga (Y) (*n* = 16)	4.0	5.0 (min 3.0; max 10.0)	6.0	6.0	7.5 (min 4.0; max 9.0)	8.0	≤0.01
Pilates (P) (*n* = 15)	4.0	5.0 (min 2.0; max 9.0)	8.0	5.0	6.0 (min 4.0; max 10.0)	9.0	≤0.01
Interval training (IT) (*n* = 15)	5.0	6.0 (min 4.0; max 8.0)	7.0	5.0	7.0 (min 4.0; max 10.0)	8.0	<0.05

Median ([IQR] (min; max) *p* value. (IQR describes the middle 50% of values when ordered from lowest to highest. Q1 is the “middle” value in the first half of the rank-ordered data set (25%) and Q3 is the “middle” value in the second half of the rank-ordered data set (75%)).

**Table 3:** Results of the Schober’s test (cm) of the four groups (target BE programme and three other programmes: yoga, Pilates and dynamic interval training) before and after the training period. The students participated voluntarily in and were randomly assigned to training programmes. The duration of each training session was one hour, twice a week for 7 weeks.

Schober’s Test (cm)							
	before			after			
	Q1	Median	Q3	Q1	Median	Q3	*p*-Value
Breathing exercises (BE) (*n* = 15)	4.5	5.0 (min 4.0; max 6.5)	6.0	5.0	5.5 (min 5.0; max 6.5)	6.0	<0.05
Yoga (Y) (*n* = 16)	3.0	4.0 (min 2.0; max 7.0)	6.0	5.0	6.0 (min 3.0; max 7.0)	7.0	≤0.01
Pilates (P) (*n* = 15)	4.0	5.0 (min 3.0; max 7.0)	6.0	6.0	6.0 (min 3.0; max 7.0)	7.0	≤0.01
Interval training (IT) (*n* = 15)	4.5	5.5 (min 3.0; max 7.0)	6.0	5.0	5.5 (min 4.0; max 8.0)	6.0	0.271

Median ([IQR] (min; max) *p* value. (IQR describes the middle 50% of values when ordered from lowest to highest. Q1 is the “middle” value in the first half of the rank-ordered data set (25%) and Q3 is the “middle” value in the second half of the rank-ordered data set (75%)).

**Table 4:** Results of the occiput-to-wall distance (OWD) test (cm) of the four groups (target BE programme and three other programmes: yoga, Pilates and dynamic interval training) before and after the training period. The students participated voluntarily in and were randomly assigned to training programmes. The duration of each training session was one hour, twice a week for 7 weeks.

Occiput-to-Wall Distance Test (cm)							
	before			after			
	Q1	Median	Q3	Q1	Median	Q3	*p*-Value
Breathing exercises (BE) (*n* = 15)	3.5	4.0 (min 3.0; max 5.0)	4.5	1.0	2.0 (min 1.0; max 3.0)	2.0	≤0.001
Yoga (Y) (*n* = 16)	1.0	3.0 (min 0.0; max 9.0)	4.5	0.0	0.0 (min 0.0; max 8.0)	0.0	≤0.01
Pilates (P) (*n* = 15)	0.0	2.0 (min 0.0; max 10.0)	5.0	0.0	0.0 (min 0.0; max 4.0)	0.0	≤0.01
Interval training (IT) (*n* = 15)	0.0	1.5 (min 0.0; max 4.5)	3.0	0.0	1.5 (min 0.0; max 6.0)	2.0	0.917

Median ([IQR] (min; max) *p* value. (IQR describes the middle 50% of values when ordered from lowest to highest. Q1 is the “middle” value in the first half of the rank-ordered data set (25%) and Q3 is the “middle” value in the second half of the rank-ordered data set (75%)). Decreased rates reflect improvement.

**Table 5:** Results of the fingertip-to-floor test (cm) of the four groups (target BE programme and three other programmes: yoga, Pilates and dynamic interval training) before and after the training period. The students participated voluntarily in and were randomly assigned to training programmes. The duration of each training session was one hour, twice a week for 7 weeks.

Fingertip-to-Floor Test (cm)							
	before			after			
	Q1	Median	Q3	Q1	Median	Q3	*p*-Value
Breathing exercises (BE) (*n* = 15)	0.0	0.0 (min 0.0; max 22.5)	5.0	0.0	0.0 (min 0.0; max 10.0)	0.0	0.056
Yoga (Y) (*n* = 16)	0.0	0.0 (min 0.0; max 23.0)	5.0	0.0	0.0 (min 0.0; max 16.0)	0.0	<0.05
Pilates (P) (*n* = 15)	0.0	1.0 (min 0.0; max 21.0)	4.0	0.0	0.0 (min 0.0; max 9.0)	0.0	<0.01
Interval training (IT) (*n* = 15)	0.0	0.0 (min 0.0; max 3.0)	0.0	0.0	0.0 (min 0.0; max 6.0)	0.0	-

Median ([IQR] (min; max) *p* value. (IQR describes the middle 50% of values when ordered from lowest to highest. Q1 is the “middle” value in the first half of the rank-ordered data set (25%) and Q3 is the “middle” value in the second half of the rank-ordered data set (75%)). Decreased rates reflect improvement.

**Table 6:** Results of the trunk side bending (lateral flexion) test (cm) to the right side of the four groups (target BE programme and three other programmes: yoga, Pilates and dynamic interval training) before and after the training period. The students participated voluntarily in and were randomly assigned to training programmes. The duration of each training session was one hour, twice a week for 7 weeks.

Trunk Side Bending (Lateral Flexion) to RIGHT Side (cm)							
	before			after			
	Q1	Median	Q3	Q1	Median	Q3	*p*-Value
Breathing exercises (BE) (*n* = 15)	40.0	42.0 (min 33.0; max 46.0)	44.0	35.0	39.0 (min 28.0; max 42.0)	40.0	≤0.001
Yoga (Y) (*n* = 16)	41.5	42.5 (min 39.0; max 52.0)	46.5	39.5	42.0 (min 35.0; max 49.0)	46.0	0.254
Pilates (P) (*n* = 15)	39.0	41.0 (min 35.0; max 48.0)	46.0	36.0	39.0 (min 35.0; max 48.0)	45.0	0.136
Interval training (IT) (*n* = 15)	41.0	45.5 (min 36.5; max 51.5)	47.0	38.0	43.0 (min 34.0; max 48.0)	45.0	≤0.01

**Table 7:** Results of the trunk side bending (lateral flexion) test (cm) to the left side in the four groups (target BE programme and three other programmes: yoga, Pilates and dynamic interval training) before and after the training period. The students participated voluntarily in and were randomly assigned to training programmes. The duration of each training session was one hour, twice a week for 7 weeks.

Trunk Side Bending (Lateral Flexion) to LEFT Side (cm)							
	before			after			
	Q1	Median	Q3	Q1	Median	Q3	*p*-Value
Breathing exercises (BE) (*n* = 15)	40.0	42.5 (min 30.5; max 48.0)	45.0	37.0	40.0 (min 29.0; max 43.0)	41.0	≤0.001
Yoga (Y) (*n* = 16)	40.5	44.0 (min 37.0; max 49.0)	46.5	39.0	42.5 (min 28.0; max 48.0)	45.0	0.156
Pilates (P) (*n* = 15)	40.0	42.0 (min 35.0; max 49.0)	46.0	37.0	40.0 (min 34.0; max 49.0)	45.0	<0.05
Interval training (IT) (*n* = 15)	42.0	45.0 (min 37.0; max 53.0)	49.0	38.5	42.5 (min 36.0; max 48.5)	47.0	<0.05

Median ([IQR] (min; max) *p* value. (IQR describes the middle 50% of values when ordered from lowest to highest. Q1 is the “middle” value in the first half of the rank-ordered data set (25%) and Q3 is the “middle” value in the second half of the rank-ordered data set (75%)). Decreased rates reflect improvement.

**Table 8:** Baseline and final pairwise differences among the four motion programmes based on Dunn’s post hoc test.

Physical Examination Tests	Compared Programs	*p* Value
Occiput-to-wall distance test before the intervention	Breathing-Interval	0.004
Occiput-to-wall distance test after the intervention	Breathing-Pilates	0.002
Occiput-to-wall distance test after the intervention	Breathing-Yoga	0.004
Occiput-to-wall distance test after the intervention	Interval-Pilates	0.038
Occiput-to-wall distance test after the intervention	Interval-Yoga	0.036
Side bending to the right after the intervention	Breathing-Interval	0.028
Side bending to the right after the intervention	Breathing-Yoga	0.030

**Table 9:** Differences between the before and after values related to the four motion programmes based on Wilcoxon signed-rank test.

	BEFORE (Median [IQR])	AFTER (Median [IQR])	*p* Value
Schober test	5 (4–6)	6 (5–6)	<0.001
Occiput-to-wall distance test	3 (1–4)	1 (0–2)	<0.001
Fingertip-to-floor test	0 (0–1)	0 (0–0)	0.001
Side bending (lateral flexion) to the right	42 (40–46)	40 (38–44)	<0.001
Side bending (lateral flexion) to the left	44 (40–46.5)	41 (38–44)	<0.001
Chest expansion	5 (4–6)	7 (6–8)	<0.001

Median ([IQR] *p* value. (IQR describes the middle 50% of values when ordered from lowest to highest. Q1 is the “middle” value in the first half of the rank-ordered data set (25%) and Q3 is the “middle” value in the second half of the rank-ordered data set (75%)).

## Data Availability

More materials were provided related to more data and the characteristics of the exercises and four motion programs. More data presented in this study and any other material related to this article and raw data can be available from the first, corresponding author on request. That data which would be used for further analysis are not publicly available now.
